# Effect of Probiotics in Breast Cancer: A Systematic Review and Meta-Analysis

**DOI:** 10.3390/biology12020280

**Published:** 2023-02-09

**Authors:** May S. Thu, Thunnicha Ondee, Tanawin Nopsopon, Izzati A. K. Farzana, Joanne L. Fothergill, Nattiya Hirankarn, Barry J. Campbell, Krit Pongpirul

**Affiliations:** 1 Center of Excellence in Immunology and Immune-Mediated Diseases, Department of Microbiology, Faculty of Medicine, Chulalongkorn University, Bangkok 10330, Thailand; 2 Department of Infection Biology & Microbiomes, Institute of Infection, Veterinary and Ecological Sciences, University of Liverpool, Liverpool L69 3GE, UK; 3 Joint Chulalongkorn University—University of Liverpool PhD Programme in Biomedical Sciences and Biotechnology, Faculty of Medicine, Chulalongkorn University, Bangkok 10330, Thailand; 4 Department of Preventive and Social Medicine, Faculty of Medicine, Chulalongkorn University, Bangkok 10330, Thailand; 5 School of Global Health, Faculty of Medicine, Chulalongkorn University, Bangkok 10330, Thailand; 6 Harvard T.H. Chan School of Public Health, Harvard University, Boston, MA 02215, USA; 7 Department of Clinical Infection, Microbiology & Immunology, Institute of Infection, Veterinary and Ecological Sciences, University of Liverpool, Liverpool L69 7BE, UK; 8 Department of International Health, Johns Hopkins Bloomberg School of Public Health, Baltimore, MD 21211, USA; 9 Bumrungrad International Hospital, Bangkok 10110, Thailand

**Keywords:** breast cancer, probiotics, prebiotics, metabolites, cytokines

## Abstract

**Simple Summary:**

Probiotics possess potential to protect against breast cancer due to their immunomodulatory activity and their ability to impact the intestinal microbiota. Experimental studies have identified key probiotic microorganisms, but their clinical role in prevention of breast cancer and the efficacy of such supplements to control chemotherapy-induced side effects is less documented. A significant number of such intervention studies have recently been published, so we, therefore, conducted a systematic review and meta-analysis of all randomized clinical trials of probiotic use in breast cancer patients and survivors, including combination prebiotic use, to provide clarity regarding actions and role/benefit for preventive and palliative care.

**Abstract:**

Probiotics may have the potential to protect against breast cancer, partly through systemic immunomodulatory action and active impact upon intestinal microbiota. Given a few clinical studies on their curative role, we conducted a systematic review of the potential effects of probiotics in breast cancer patients and survivors of breast cancer, aiming to support further clinical studies. A literature search was performed using PubMed, Embase, and the CENTRAL databases from inception through to March 2022. A total of eight randomized clinical trials were identified from thirteen articles published between 2004 and 2022. We evaluated quality-of-life measures, observed bacterial species and diversity indices, probiotic-related metabolites, inflammatory biomarkers, and other responses in breast cancer patients and survivors. Results were synthesized qualitatively and quantitatively using random-effects meta-analysis. Different probiotics supplements utilized included *Lactobacillus* species alone (Lacto), with or without estriol; probiotic combinations of *Lactobacillus* with *Bifidobacterium* (ProLB), with or without prebiotic fructooligosaccharides (FOS); ProLB plus *Streptococcus* and FOS (ProLBS + FOS); and ProLB plus *Enterococcus* (ProLBE). We found that use of ProLBS with FOS in breast cancer patients and use of ProLBE in survivors of breast cancer show potential benefits in countering obesity and dyslipidemia. ProLBS with FOS use decreases pro-inflammatory TNF-α in breast cancer survivors and improves quality of life in those with breast-cancer-associated lymphedema. Supplementing probiotics capsules (10^9^ CFU) with a prebiotic and using an intake duration of 10 weeks could provide a better approach than probiotics alone.

## 1. Introduction

Probiotics can be defined as live microorganism preparations (particularly bacteria or yeasts) that, when administered to a host, confer health benefits [1]. Their biological actions were first postulated over a century ago by Russian scientist and Nobel Prize winner Elie Metchnikoff [1]. Scientific evidence on efficacy and safety of probiotics within food, and those provided as supplements, was reviewed by a joint expert panel of the Food and Agriculture Organization of the United Nations and the World Health Organization (FAO/WHO) [2]. More detailed research has been conducted in recent decades on their use for treatment and prevention of gastrointestinal diseases [3,4]. Different mechanisms of their benefit to human health have been identified, including maintenance of a healthy intestinal microbiota community structure, enhancement of mucosal barrier function and defense against pathogen invasion of the intestinal epithelium, and beneficial immunomodulatory activity [5,6].

Lactobacilli, lactic acid-producing bacteria that are primarily obtained through consumption of fermented dairy products such as yogurt, are the most commonly employed strains used for probiotic supplements, either added to the diet or in capsular form [7]. It has been estimated that up to 30% of probiotic strains survive for a few hours within the GI tract, although this is dependent on several factors, including probiotic species/strain of choice, acid/pH tolerance, and ability to establish themselves as a significant presence within the host microbiota [8,9,10]. Probiotics have been discovered to suppress β-glucuronidase-producing bacteria that have the potential to metabolize pre-carcinogens to active carcinogens known to contribute to colon carcinogenesis [11].

Numerous in vitro and in vivo studies have demonstrated that probiotics can be effective at controlling growth of cancer cells [12,13]. Significant protective benefits against colon cancer are most likely due to direct biological interaction with the colonic epithelium [14], with strains, such as *Lactobacillus rhamnosus* GG, shown to suppress proliferation and promote apoptosis in colon cancer cell lines [15]. A prospective study of the EPIC-Italy cohort via a dietary questionnaire revealed that yogurt intake (containing high counts of viable *Streptoccocus thermophilus* and *Lactobacillus delbrueckii* subsp. *bulgaricus*) was inversely associated with risk of colorectal cancer [16]. In addition, an oral *Lactobacillus casei* preparation is effective in preventing recurrence of superficial bladder cancer [17].

Prebiotics, oligosaccharides that are non-digestible but fermentable, also have the potential to alter composition and activity of intestinal microbiota to benefit host health [18]. Similar to probiotics, prebiotics also possess significant anti-carcinogenic activity and are a promising tool for use in GI cancer prevention and/or therapy [19]. Use of ‘synbiotics’, a combination of one or more probiotics with prebiotics, is also an option for managing gut microbiota and alleviating side effects of cancer therapies [20]. In vivo evidence has shown that prebiotic polysaccharides can prevent azoxymethane/dextran-sodium-sulfate-induced colorectal cancer in C57BL/6 mice [21]. In a clinical trial involving 140 perioperative colorectal cancer patients (90 men and 50 women aged 40–75 years), daily oral intake of 30 g prebiotic supplement containing fructooligosaccharides (FOS, 25%), xylooligosaccharides (25%), polydextrose (25%), and resistant dextrin (25%) for 1 week showed significant positive effects on immune status of patients in both preoperative and postoperative periods with CRC [22]. In addition, intake of prebiotics increased prevalence of four commensal microbiota in these individuals, *Bacteroides, Bifidobacterium, Escherichia-Shigella*, and *Enterococcus* [22].

In recent years, the prevalence of female breast cancer has increased markedly, affecting women more than any other type of cancer [23]. The immune system in cancer patients is known to be impaired due to primary disease and following cancer therapy [24,25]. There is increasing evidence that probiotics can effectively support management of cancer cases [12,26,27]. As an example, probiotics containing *Lactobacillus rhamnosus* LC705 and *Propionibacterium freudenreichii* subsp. *shermaniis* significantly lowered risk of liver cancer, reducing intestinal absorption of pro-carcinogenic aflatoxins [28].

The significance of the human intestinal microbiome in etiology of breast cancer is emphasized by studies linking gut microbiota dysbiosis with high risk of developing breast cancer [29]. Additionally, the intestinal microbiota participates in metabolism of isoflavones, which, by possessing anti-inflammatory, antioxidant, antiangiogenic, and phytoestrogenic activities, contributes to breast cancer pathophysiology [24,25]. This is particularly so for estrogen-dependent breast cancers because of their role in modulating non-ovarian estrogen levels via enterohepatic circulation [30,31,32].

To date, there have been numerous studies showing the anti-cancer effects of probiotics, particularly, but not exclusively, using *Lactobacillus* spp. on several breast cancer cell lines and in xenograft models of breast cancer [33]. Case-control studies also support the role of *Lactobacillus* species in breast cancer [34,35]. A Japanese population-based case-control study, comprising 306 breast cancer cases and 662 controls, concluded that regular consumption of *Lactobacillus casei* Shirota and soy isoflavones since adolescence was significantly associated with decreased risk of breast cancer in women [35].

To support in vitro, in vivo, and case-control studies evaluating the effects of probiotics and prebiotics on breast cancer treatment and prevention, more clinical intervention studies are warranted. However, few studies to date have been conducted, each utilizing different probiotics (species/strain, combination regimens, and duration of use) with or without prebiotics, and their role/benefit for preventive and palliative care is even less documented. Therefore, the main objective of this study was to conduct a systematic review of use of probiotics in breast cancer patients and survivors to explore various outcomes of any probiotic treatment (such as quality of life, alteration in bacterial profile, and diversity and changes in different metabolites in the host) to provide clarity regarding their role/benefit for preventive and palliative care. Our observations may also support further research on alternative or combinatorial use of probiotics in breast cancer.

## 2. Materials and Methods

### 2.1. Protocol Registration

The study was registered on PROSPERO (www.crd.york.ac.uk/prospero accessed on 28 July 2022); ID CRD42022349686 (accessed on 7 August 2022).

### 2.2. Literature Search, Study Selection, and Data Extraction

The systematic literature review and meta-analysis were carried out following the PRISMA declaration standards (see Supporting Information File S1) [36]. The PICOs (Population, Intervention, Comparison or Controls, and Outcome) framework served as the basis for inclusion and exclusion criteria for the study [37,38]. Regarding participants, intervention, and controls, inclusion was limited to randomized intervention studies evaluating any probiotic treatment in patients and survivors of breast cancer, with or without any active or placebo control. Inclusion was also limited to those studies published in the English language. Studies that had not exclusively used human participants (i.e., in vitro research and animal studies), review articles, procedures, letters, editorials, commentaries, recommendations, and guidelines were all excluded, as well as any study that had not been peer-reviewed. Literature was sourced from three different databases: PubMed (https://pubmed.ncbi.nlm.nih.gov/ accessed on 3 March 2022), Embase (www.embase.com accessed on 3 March 2022), and Cochrane Library (www.cochranelibrary.com accessed on 3 March 2022). This was conducted using a full search term strategy, as detailed in Supporting Information File S2. Sourced publications identified from these databases up until 3 March 2022 were imported into the Covidence platform (www.covidence.org/; accessed on 31 October 2022) for systematic screening.

In an initial screen of all imported articles, four of the authors (I.A.K.F., M.S.T., T.N., and T.O.) independently evaluated each study for consideration of inclusion within the systematic review. Discrepancies in selection for inclusion were settled through group discussion and consensus agreement at each stage. For data extraction, all pertinent full-text documents were obtained, with information within the text, tables, and all figures scrutinized. Data extraction was performed by three of the authors (I.A.K.F., M.S.T., and T.O.) for the following variables: (1) authors, year of publication, study type, number and age range of study participants, probiotic regimens evaluated, including dose and duration of treatment, and the country that implemented the study; (2) patient demographics, anthropometric parameters (weight, body mass index (BMI), waist circumference, etc.), and characteristics, such as stage and hormonal status of breast cancer; (3) related characteristics/outcomes, including different changes in metabolites, cytokines (measured in serum and urine), and high-sensitivity CRP (hs-CRP); and (4) microbial diversity.

### 2.3. Risk of Bias Analysis

The independent team (I.A.K.F., M.S.T., and T.O.) also assessed risk of bias (ROB) in the retrieved intervention studies using Cochrane Risk of Bias tool 2.0 (ROB2; https://methods.cochrane.org/risk-bias-2; accessed on 31 October 2022) [39,40]. The tool was used to assess the following domains: bias arising from the randomization process; bias due to deviations from intended intervention; bias due to missing outcome data; bias in measurement of the outcome; and bias in selection of the reported result(s). Any differences of opinion were settled through consensus. If data were insufficient, the associated authors were emailed and a two-week response period was allowed for them to react. If there was no answer, the situation was handled using the information at hand and any discrepancies were worked out through conversation.

### 2.4. Subgroup Analysis

Analysis was conducted in the following subgroups: probiotic supplements (*Lactobacillus* only, combination of probiotics (with or without prebiotics)) and intake duration.

### 2.5. Statistics

For intervention studies, mean differences (MD) along with 95% confidence intervals (CI) between groups were indicated for probiotic-related outcomes. Statistical heterogeneity was represented utilizing *I^2^* statistics [39]. For clinical, methodological, and statistical heterogeneity, random effects meta-analysis by DerSimonian and Laird method was utilized by RevMan 5, v.5.4.1 (https://training.cochrane.org/online-learning/core-software/revman/; accessed on 31 October 2022). Following standard 4.2, conduct a qualitative synthesis, in Chapter 4 of ‘*Finding What Works in Health Care: Standards for Systematic Reviews*’, we provided qualitative analysis of trials and their results [40].

## 3. Results

### 3.1. Study Selection

**Figure 1:**
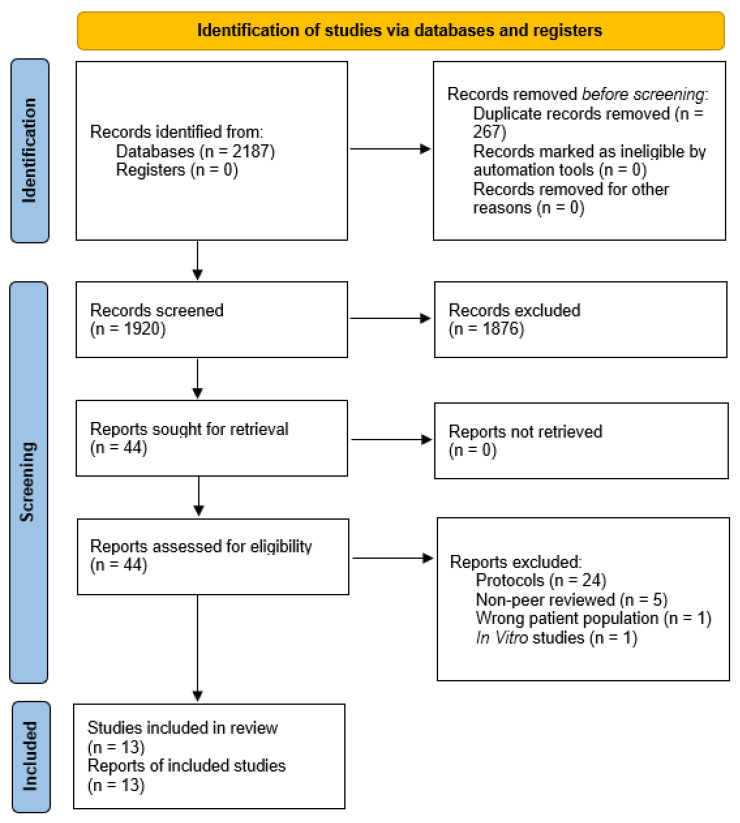
Flow diagram for identification of studies in the systematic review.

From a total of 2187 articles retrieved, 267 duplicates were eliminated before screening. Following review of titles and abstracts of the remaining 1920 studies, 1876 papers were excluded and 44 articles remaining were retrieved for full-text screening and their eligibility assessed for meta-analysis. Of these, thirty-one publications were disregarded; one studied non-breast cancer patients, one was an in vitro study, twenty-four were protocol papers, and five non-peer-reviewed articles. Finally, thirteen intervention studies, from eight trials that enrolled five-hundred-seventy-one people across a research period from 2003 to 2019, were included in the systematic review and meta-analysis (Figure 1).

### 3.2. Study Characteristics

The thirteen included studies, published between 2004 and 2022, were conducted across six different nations (Austria, Belgium/Germany, China, Iran, and the USA). Eight trials were identified: one randomized crossover trial and seven randomized controlled trials, where participants were randomly assigned to a control group, placebo group, or intervention group to reduce allocation bias. Participants across all studies ranged in age from 18 to 75. Various probiotic regimens were examined: Lactobacillus spp. alone (Lacto) or Lactobacillus with Bifidobacterium (ProLB), and Streptococcus (ProLBS) or Enterococcus (ProLBE) with or without prebiotic FOS supplementation at various doses in breast cancer patients and/or survivors. Length of treatment ranged from 2–10 weeks (Table 1).

### 3.3. Subject Characteristics

In total, 571 participants were involved in the review; 51% were assigned to the intervention (probiotics and/or prebiotics) group, 38% were assigned to the placebo group, and 11% to the control group. The average age of the participants receiving the intervention, the placebo, and within the control group were 51.01 (SD = 8.78), 51.33 (SD = 8.26), and 53.24 (SD = 3.55), respectively. A total of 373 study participants (65%) were recorded as having a BMI ≥ 25. This included 63% of the group receiving probiotics, 58% receiving placebo, and all identified control subjects. The review included patients with breast cancer in stages I through III, where cancer still occurred or patients had recovered, with a higher percentage of Stage II cases. Three-quarters of participants in each assignment were ER-positive, more than two-thirds of cases were PR positive, and more than 68% were HER2-negative. Iran accounted for around half of the participants in both the intervention and placebo groups among the various study regions (Table 2).

### 3.4. Risk of Bias

**Figure 2:**
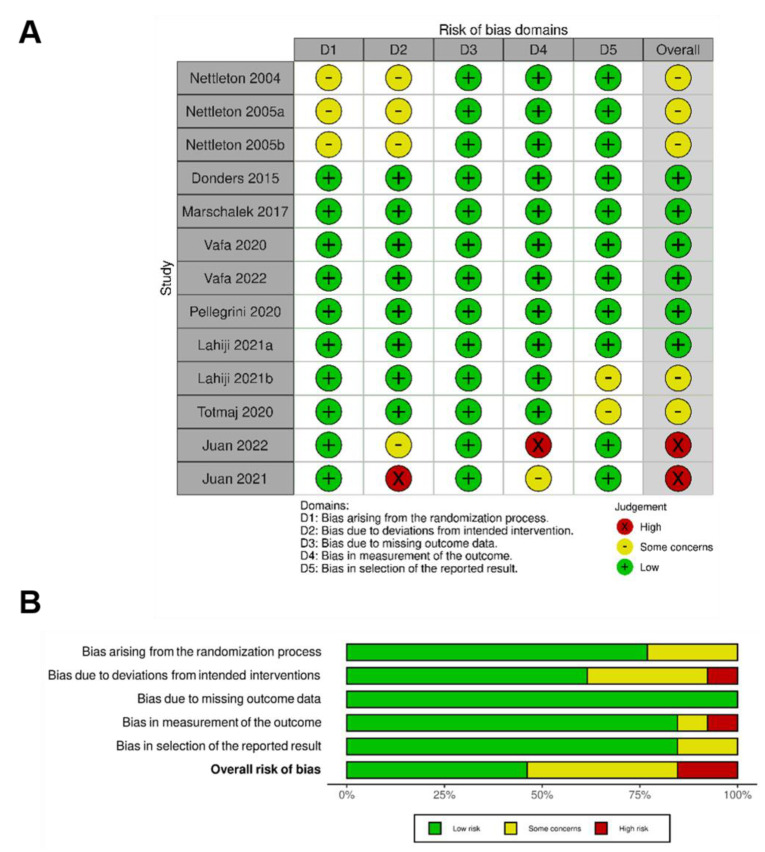
Risk of bias (ROB) analysis highlighting results in all domains examined within the 13 identified studies (**A**) and overall risk of bias for included studies (**B**).

Two of the thirteen included studies [52,53] were identified as having overall high ROB, five studies [41,42,43,48,51] were interpreted as having some concerns (particularly in either ROB domains 1 and 2 or domain 5), and six studies [44,45,46,47,49,50] were identified with a low ROB for all domains (Figure 2A). The overall summary of risk of bias (ROB) of the thirteen included studies appears in Figure 2B.

### 3.5. Qualitative Analysis

By demographic, intervention, control, sample type, and summary of results, all the collected studies were qualitatively assessed (Table 3). Generally, the table visualization covers the effects of different probiotics profile with or without prebiotics on different outcomes, such as phytoestrogen concentrations, estrogen profile, vaginal microbiota, inflammatory markers and cytokines, abundance of bacterial communities and their diversity indexes, and other metabolic and anthropometric parameters.

### 3.6. Probiotics and Prebiotics

One study made use of *Lactobacillus* alone; one used *Lactobacillus* and ultra-low-dose 0.03 mg estriol (E3); the others explored use of probiotic combination regimens that included both *Lactobacillus* and *Bifidobacterium* and either *Streptococcus or Enterococcus*, along with addition of prebiotic FOS. Probiotics containing *Lactobacillus* and *Bifidobacterium* (ProLB) were utilized in two trials. *Lactobacillus, Bifidobacterium*, and *Streptococcus* (ProLBS) were included in three trial protocols. Overall, four trials included FOS as a prebiotic in combination with the probiotic treatment. Intervention comprising *Lactobacillus, Bifidobacterium*, and *Enterococcus* (ProLBE) was employed in a single trial but did not include any prebiotic supplementation. For detailed information, see Table 1 and Table 3.

**Figure 3:**
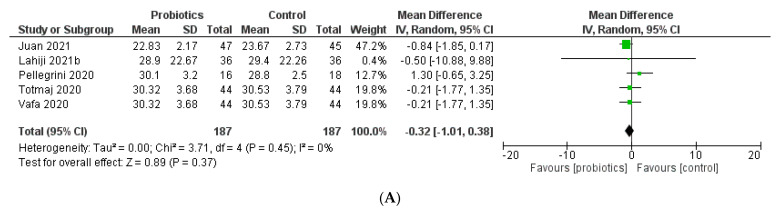

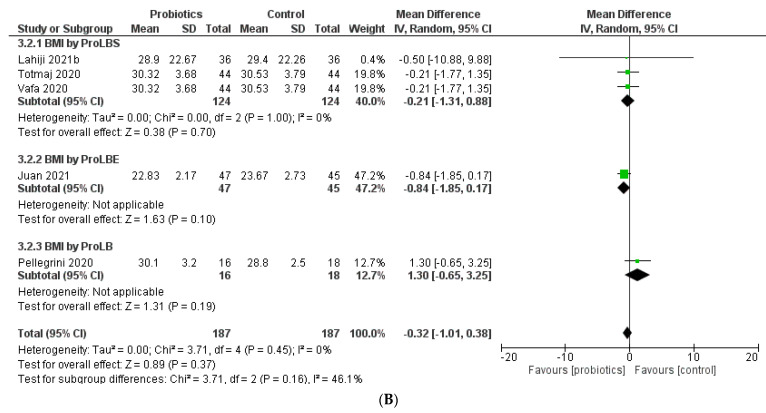
Meta-analysis forest plot for (**A**) body mass index (BMI) and (**B**) BMI by probiotic type.

**Figure 4:**
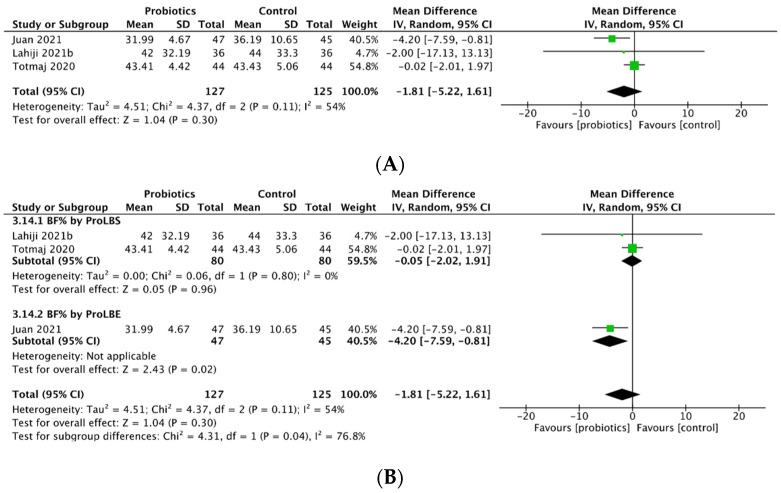
Meta-analysis for (**A**) percentage change in body fat (BF%) percent and (**B**) BF% by probiotic type.

**Figure 5:**
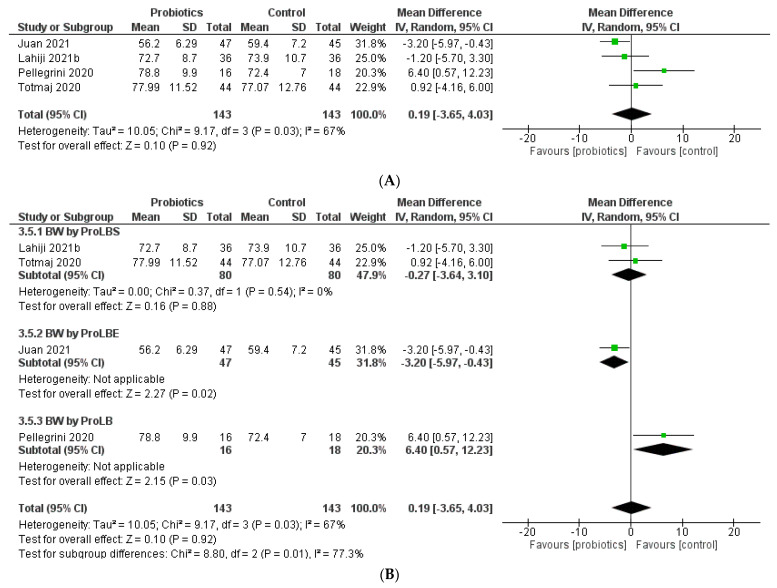
Meta-analysis of body weight (**A**) and body weight by probiotics type (**B**).

**Figure 6:**
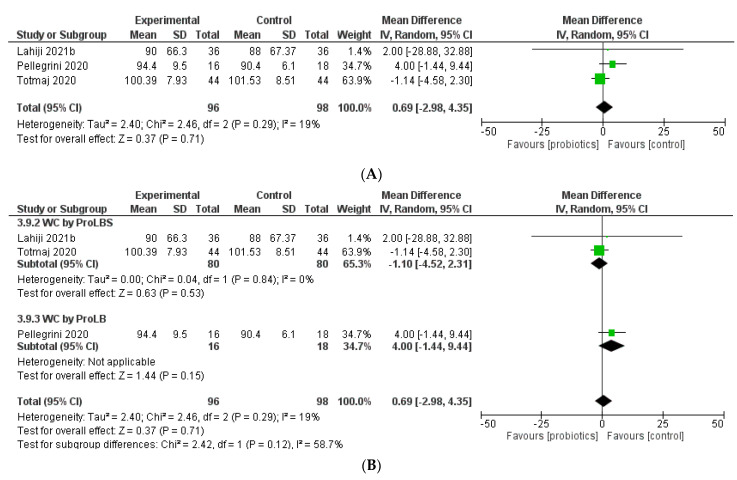
Meta-analysis for waist circumference (WC) (**A**) and WC by probiotics type (**B**).

**Figure 7:**
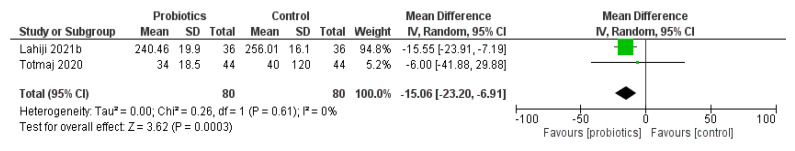
Meta-analysis for tumor necrosis factor-alpha (TNF-α).

**Figure 8:**
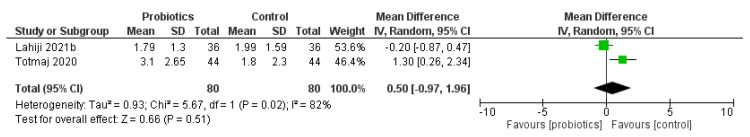
Meta-analysis for high-sensitivity C-reactive protein (hs-CRP).

**Figure 9:**
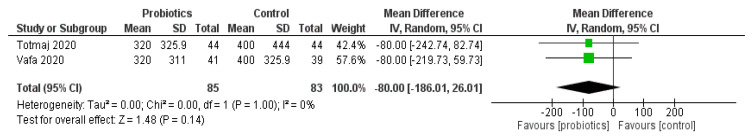
Meta-analysis of edema volume.

We found that the anthropometric parameters, such as BMI, waist circumference (WC), BF%, and edema volume, were reduced after probiotic intervention rather than body weight (BW). TNF-α and hs-CRP were not reduced with intervention (Figure 3, Figure 4, Figure 5, Figure 6, Figure 7, Figure 8 and Figure 9). Supplementing with prebiotics improved BMI according to sensitivity analysis (SMD = −0.05; 95% CI: −0.26 to 0.17; *p* = 0.66), but other anthropometric measurements did not change. Additionally, compared to 8-week interventions, probiotic use for 10 weeks raised BMI (SMD = −0.06; 95% CI: −0.30 to 0.19; *p* = 0.65). However, it is challenging to evaluate for a 3-week intervention study because the study utilized a greater dose over a shorter time frame and no heterogeneity can be calculated for a single trial (see Table 4).

### 3.7. Body Mass Index

In five studies, BMI was assessed both before and after probiotic and placebo treatments (Figure 3). Overall, the meta-analysis showed that probiotics decreased the BMI of breast cancer patients and survivors in comparison to placebo (MD = −0.32; 95% CI: −1.01 to 0.38; *p* = 0.37). However, this difference was not statistically significant. ProLBS (MD = −0.21; 95% CI: −1.31 to 0.88; *p* = 0.70) and ProLBE (MD = −0.84; 95% CI: −1.85 to 0.17; *p* = 0.10) both reduced BMI after treatment according to subgroup analysis by different probiotic regimens.

### 3.8. Percentage Change in Body Fat

Three studies assessed percentage change in body fat (BF%) after probiotics intervention. Use of probiotics reduced BF% in both breast cancer patients and survivors (MD = −1.81; 95% CI: −5.22 to 1.61; *p* = 0.30; Figure 4A). Subgroup analysis demonstrated that ProLBE supplements significantly reduced the elevation of BF% in breast cancer patients (MD = −4.20; 95% CI: −7.59 to −0.81; *p* = 0.02) while a smaller decrease in BF% occurred in breast cancer survivors with use of ProLBS capsules (Figure 4B).

### 3.9. Body Weight

An overall increase following intervention was reported for body weight (BW) levels in four studies (MD = 0.19; 95% CI: −3.65 to 4.03; *p* = 0.88) (Figure 5). A significant change in effect of probiotics was described in the subgroup analysis of ProLBE use in breast cancer patients (MD = −3.20; 95% CI: −5.97 to −0.43; *p* = 0.02).

### 3.10. Waist Circumference

An overall estimate of three studies indicated no significant elevation in waist circumference (WC) in the intervention groups (MD = 0.69; 95% CI: −2.98 to 4.35; *p* = 0.71) (Figure 6). The subgroup analysis showed a decrease in WC of breast cancer survivors (MD = −1.10; 95% CI: −4.52 to 2.31; *p* = 0.53) who used ProLBS; however, ProLB did not provide any improvement in WC (MD = 4; 95% CI: −1.44 to 9.44; *p* = 0.15).

### 3.11. Tumor Necrosis Factor-Alpha

The meta-analysis on tumor necrosis factor-alpha (TNF-α) revealed a significant improvement following intervention with ProLBS capsules plus FOS (MD = −15.06; 95% CI: −23.20 to −6.91; *p* = 0.0003); see Figure 7.

### 3.12. High-Sensitivity C-Reactive Protein

Analysis of two studies including hs-CRP data in breast cancer survivors indicated that intervention with ProLBS plus FOS did not cause any overall alterations to hs-CRP levels detected (MD = 0.5; 95% CI: −0.97 to 1.96; *p* = 0.51). Furthermore, the between-study heterogeneity was significantly high (*I*^2^ = 82%, *p* = 0.02); see Figure 8.

### 3.13. Edema Volume

In the meta-analysis of edema volume observed in breast cancer survivors, two studies indicated some improvement with ProLBS plus FOS intervention (MD = −80.00; 95% CI: −186.01 to 26.01; *p* = 0.14) but did not achieve statistical significance (Figure 9).

## 4. Discussion

The overall systematic review and meta-analysis indicated that BMI and BF% decreased in the intervention and BW did not undergo any changes. These findings support the notion that probiotics may help to reduce obesity and dyslipidemia [54]. Specifically, ProLBE had a significant protective role in reducing BF% and BW in breast cancer patients. However, ProLB did not provide any changes in BMI (MD = 1.30; 95% CI: −0.65 to 3.25; *p* = 0.19), WC (MD = 4; 95% CI: −1.44 to 9.44; *p* = 0.15), or BW (MD = 6.4; 95% CI: 0.57 to 12.23; *p* = 0.03). In addition, our study identified that ProLBS in combination with prebiotic FOS supplementation effected a reduction in level of circulating TNF-α [MD = −15.06; 95% CI: −23.20 to −6.91; *p* = 0.0003] in the population of breast cancer survivors. TNF-α is a key pro-inflammatory cytokine in the etiology of breast cancer, with the TNF-TNFR2 axis being cited as highly important [55]. TNF-α has been shown to drive increased proliferation of breast cancer cells and suppression of the host immune response against a developing tumor [55,56]. Increased tissue levels of TNF-α observed in breast cancer are also associated with higher-grade tumors, increased risk of metastasis, poor treatment outcomes, and low chance of recovery from the disease [55,56]. Probiotics, such as *Lactobacillus*, *Bifidobacterium,* and *Streptoccoccus* spp., are known to inhibit TNF-α transcription and release from many epithelial cell types through targeted suppression of activity of key cell pro-inflammatory signal pathways [57]. Given the significant role of TNF-α, use of probiotic supplementation would appear warranted to reduce cancer severity and/or symptoms and provide improvement in prognosis for both breast cancer patients and survivors.

High-sensitivity C-reactive protein (hs-CRP) is also a simple-to-measure biomarker that can be raised in both acute and chronic diseases and represents systemic inflammation, infection, or tissue damage in the body [58]. No significant differences were found among breast cancer survivors, meaning that ProLBS in combination with FOS may have no protective effects on the inflammatory marker of breast cancer cases.

Lymphedema issues may persist for months, or even years, following breast cancer therapy [59]. Breast-cancer-related lymphedema (BCRL) has a reported incidence of 21.4% [60] and is characterized by swelling, heaviness, pain, restrictions on how much an individual may use their limbs, and lower quality of life [61]. Here, the meta-analysis showed that ProLBS decreased edema volume experienced by breast cancer survivors, although this effect was not determined to be statistically significant.

We identified an intervention study utilizing a ProLBS and FOS combination regimen that assessed quality of life in lymphedema patients using the Lymphedema Life Impact Scale (LLIS) questionnaire [46]. This questionnaire covers physical, psycho-social, and functional activities, where the total and each subscale score are a percentage ranging from 0 to 100 in which a higher percentage of impairment indicates lower quality of life due to lymphedema. We noted that, within this study, their probiotic group had a 39% improvement in total LLIS (median = −39.53, IQR = 50.2), 42% betterment in physical LLIS (median = −42.10, IQR = 62.5), and a 36% improvement in functional LLIS (median = −36.36, IQR = 60), being significantly different compared to the placebo group [46].

Additionally, we identified that probiotics including *Lactobacillus* and *Bifidobacterium* spp. (ProLB), used every day for two months as part of a 4-month Mediterranean diet, had a substantial impact on bacterial species that were observed (*p* = 0.01) and alpha-diversity (*p* = 0.004) [49]. At the end of the intervention, *Escherichia* levels were greater and Clostridiales levels were lower in the intervention group at baseline, and the probiotic-treated group had a large rise in both *Ruminococcus* assigned to families Lachnospiraceae and *Eubacterium* and a significant decrease in *Bacteroides* and *Butyricicoccus* (*p* ≤ 0.05) [49]. In the intervention group, the Bacteroidetes-to-Firmicutes ratio considerably decreased, whereas it increased in the control group (*p* = 0.004). Compared to Mediterranean diet alone, probiotic supplementation had a positive impact on gut microbiota diversity.

Qualitative analysis within our review (Table 3) has provided detailed findings in all the probiotics-related trials in breast cancer patients and survivors. Furthermore, the meta-analysis covered anthropometric measurements, inflammatory cytokines, and edema volume. In the crossover trial conducted by Nettleton and colleagues [41,42,43], no significant differences in plasma phytoestrogen levels were found in breast cancer survivors and controls; however, lower levels of most phytoestrogens, especially genistein, in the survivors revealed probable differences in gut microbiota that may alter phytoestrogen metabolism and impact cancer risk. No variation in equol level between the survivors and controls at baseline and during consumption of soy and milk diets is concordant with a study by Adlercreutz et al. [62]. A subsequent study by Nettleton and colleagues also revealed no significant differences in 2-hydroxy estrone (2-OHE), 16-hydroxy estrone (16OHE_1_), and 2:16OHE_1_ but lower 2:16OHE_1_ in breast cancer survivors [42]. Furthermore, no differences in sex-hormone-binding globulin (SHBG), which is a hormonal factor and may influence estrogen metabolism by the liver, were found in the postmenopausal survivors, but soy protein tended to decrease SHBG concentrations relative to milk protein [43]. According to these studies, consumption of soy protein isolate, probiotic supplementation, or equol producer status did not affect levels of reproductive hormones and neither did presence of breast cancer or equol producer status change the effects of soy protein isolate or probiotic supplementation.

Research has revealed that the fecal microbiota of breast cancer patients differs from that of healthy individuals, being less diverse [29]. Probiotics, used to restore beneficial gut microbiota, are considered to be safe, and the right supplement preparation and dose may help in treatment of breast cancer. Here, we have identified that taking ProLB (a sachet of 4 × 10^9^ CFU daily) for 8 weeks, as part of a 4-month Mediterranean diet plan, positively influences gut microbiota composition, illustrating the potential to act therapeutically against breast cancer. Key RCTs also support use of ProLBS (a capsule of 10^9^ CFU daily, for 8 or 10 weeks) together with prebiotic FOS supplements (38.5 mg daily) to provide significant decrease in pro-inflammatory, pro-oncogenic TNF-α [48,51], and significant improvement in quality of life of patients with breast-cancer-related lymphedema [46]. Furthermore, three capsules (0.84 g) of ProLBE, twice daily for 3 weeks, have proven highly beneficial in preventing weight gain and obesity [52,53], key factors linked to poor disease outcomes in breast cancer patients [63].

It is important to acknowledge that two of the identified clinical trials, covering three studies [45,52,53], focused on breast cancer patients receiving chemotherapy using probiotic supplements Lacto and ProLBE. Due to the limited number of available studies meeting inclusion criteria, our meta-analysis is a combination of these studies including both breast cancer patients and breast cancer survivors; hence, they are not differentiated as independent groups. Since the included trials also provided a mix of multiple several hormonal types of breast cancer patients and reported only overall results, it was limited to performing subgroup analysis for breast cancer sub-types in clinical practice. In addition, only one of the eight included trials included information regarding adverse events/negative impacts of their interventions. The trial conducted by Donders and colleagues [44] reported safety data within the main results of an earlier phase I pharmacokinetic study [64], detailing no serious adverse effects but that adverse events of mild to moderate severity were noted, with over 60% likely related to the study medication.

## 5. Conclusions

To conclude, this systematic review and meta-analysis emphasizes the effects of different probiotics/prebiotics supplements on decreasing several key anthropometric parameters and regarding key microbial changes. Following from this review, it is anticipated that there will be further clinical trials of probiotics on patients and survivors of breast cancer that will address improvement in quality of life of individuals and investigate synergistic benefits with their cancer treatment.

## Figures and Tables

**Table 1:** Baseline characteristics within the identified studies.

Study Number	First Author, Year (Reference)	Country	Study Type	Participant Numbers (*n*)	Age Range (Years)	Probiotic Regimen	Dose	Duration
1	Nettleton, 2004 [41]	USA	Randomized crossover trial	40	36–72	ProLB + FOS	3 capsules (10^9^ CFU)/15–30 mg FOS before breakfast	6 weeks
2	Nettleton, 2005a [42]							
3	Nettleton, 2005b [43]							
4	Donders, 2015 [44]	Belgium/Germany	Randomized trial	16	52–63	Lacto + ultra-low dose 0.03 mg estriol (E3)	1 tablet (Gynoflor^®^) daily followed by maintenance therapy for 8 weeks	4 weeks
5	Marschalek, 2017 [45]	Austria	Randomized placebo-controlled trial	22	18–45 *	Lacto	1 capsule (2.5 × 10^9^ CFU) daily, twice/day	2 weeks
6	Vafa, 2020 [46]	Iran	Parallel, randomized, placebo-controlled trial	135	50–57	ProLBS + FOS	1 capsule (10^9^ CFU)/38.5 mg FOS daily	10 weeks
7	Vafa, 2022 [47]	Iran	Randomized clinical trial	88	35–73	ProLBS + FOS	1 capsule (10^9^ CFU)/38.5 mg FOS daily	10 weeks
8	Totmaj, 2020 [48]							
9	Pellegrini, 2020 [49]	Iran	Randomized open-label trial	34	<70 *	ProLB	1 sachet (4 × 10^9^ CFU) daily	2 months
10	Lahiji, 2021a [50]	Iran	Randomized placebo-controlled	76	50–75	ProLBS + FOS	1 capsule (10^9^ CFU)/38.5 mg FOS daily	8 weeks
11	Lahiji, 2021b [51]							
12	Juan, 2022 [52]	China	Randomized placebo-controlled trial	160	28–63	ProLBE	3 capsules (0.84 g) per time, twice/day	3 weeks
13	Juan, 2021 [53]			100				

Abbreviations: Lacto, *Lactobacillus* spp. alone; ProLB, probiotics comprising *Lactobacillus* and *Bifidobacterium*; ProLBS, probiotics comprising *Lactobacillus*, *Bifidobacterium*, and *Streptococcus*; ProLBE, probiotics comprising *Lactobacillus*, *Bifidobacterium*, and *Enterococcus*; FOS, fructooligosaccharides. * inclusion criteria available only.

**Table 2:** Demographic characteristics of participants.

Characteristics	Intervention	Placebo	Control
Total numbers, *n* (% total)	290 (51%)	218 (38%)	63 (11%)
Age, mean (± SD)	51.01 (8.78)	51.33 (8.26)	53.24 (3.55)
BMI (kg/m^2^), *n* (%)			
< 25	96 (33%)	80 (37%)	0
≥ 25	183 (63%)	127 (58%)	63 (100%)
Unknown	11 (4%)	11 (5%)	0
Breast cancer stage, *n* (%)			
Stage I	44 (21%)	19 (16%)	27 (20%)
Stage II	125 (59%)	79 (66%)	73 (55%)
Stage III	44 (21%)	21 (18%)	32 (24%)
ER status, *n* (%)			
Positive	72 (76%)	71 (76%)	28 (74%)
Negative	23 (24%)	22 (24%)	10 (26%)
PR status, *n* (%)			
Positive	70 (74%)	59 (66%)	28 (68%)
Negative	25 (26%)	30 (34%)	13 (32%)
HER2 status, *n* (%)			
Positive	34 (19%)	38 (22%)	6 (32%)
Negative	141 (81%)	135 (78%)	13 (68%)
Country, *n* (%)			
USA	40 (14%)	0	0
Belgium/Germany	16 (6%)	0	0
Austria	11 (4%)	11 (5%)	0
Iran	143 (49%)	127 (58%)	63 (100%)
China	80 (28%)	80 (37%)	0

Abbreviations: ER, estrogen receptor; PR, progesterone receptor; HER2, human epidermal growth factor receptor 2.

**Table 3:** Qualitative analysis for all the included studies amongst the identified trials.

#	Author, publication year (reference)	Population	Intervention	Control	Sample Type	Findings
1	Nettleton, 2004 [41]	Breast cancer (BC) survivors	1. Diet+Soy protein isolate (S); 2. Diet+S+Probiotics (S+P) 3. Diet+Milk protein isolate (M); 4. Diet+M+Probiotics (M+P) *Four 42 d diet plan in random order	-	Plasma, 24 hr urine	1. No changes in plasma phytoestrogen between groups. 2. No changes between S and S+P diets due to plasma phytoestrogen levels and number of equol producers. 3. Probiotic supplement does not generally affect plasma isoflavones.
2	Nettleton, 2005a [42]					1. Soy consumption tended to increase urinary 2-OHE (*p* = 0.07) and 16α-OHE_1_ (*p* = 0.11) but had no effect on urinary 2:16OHE_1_. 2. Soy consumption increased 2:16OHE_1_ only in women who are equol producers.
3	Nettleton, 2005b [43]					1. Soy, probiotic supplements, or equol producer status had no impact on hormone levels. 2. Neither presence of cancernor or equol producers changed the effects of soy or probiotics.
4	Donders, 2015 [44]	Postmenopausal BC survivors on aromatase inhibitors with severe atrophic vaginitis	Vaginal use of 0.03 mg estriol and lactobacilli (1 tablet of Gynoflor^®^ for 28 d) combination	-	Vaginal smear	1. Lactobacillary grades (*p* < 0.001) and aerobic vaginitis (*p* < 0.01) improved during treatment. 2. Leukocytes (*p* < 0.01) and parabasal cells (*P*_trend_ < 0.01) dropped at the final visit. 3. *Candida* may develop soon after its use but rapidly disappears again upon their prolonged use.
5	Marschalek, 2017 [45]	Postmenopausal BC patients receiving chemotherapy, with vaginal atrophy and an intermediate vaginal microbiota (Nugent score 4–6)	Twice daily oral capsules for 2 weeks	Oral placebo having lactose	Vaginal smear	1. Observed a positive influence on vaginal microbiota in 63% women in the intervention group and 36% women in the control group. 2. There was a shift in Nugent score towards normal microbiota levels in the intervention group and significant deterioration in the score in the control group.
6	Vafa, 2020 [46]	BC survivors with breast-cancer-related lymphedema (BCRL)	A calorie-restricted diet plus a synbiotic (CRS) daily for 10 weeks	Diet plus a placebo (CRP) and control	Body fluid	1. A decrease in the total quality-of-life score (*p* = 0.004), and its psychosocial (*p* = 0.022) and functional (*p* = 0.002) domain scores 2. A decrease in edema volume (*p* = 0.002) and BMI (*p* < 0.001) in comparison to controls.
7	Vafa, 2022 [47]	Overweight or obese BC survivors with BCRL	Low-calorie diet (LCD) plus a synbiotic daily for 10 weeks	LCD plus a placebo	Serum	1. Had beneficial effects on increasing serum TGF-β, IL-10, and adiponectin levels in women with BCRL, but no significant differences. 2. Edema volume decreased in the synbiotic group. 3. BW, BMI, BF%, and WC decreased in both groups.
8	Totmaj, 2020 [48]					1. A significant reduction in leptin (*p* = 0.003) and TNF-α (*p* = 0.039) between the groups. 2. No significant effects in hs-CRP (*p* = 0.55) and IL-1β (*p* = 0.118) between study groups.
9	Pellegrini, 2020 [49]	Overweight BC survivors	Mediterranean diet for 4 mo. + Probiotics for first 2 mo.	Mediterranean diet for 4 mo. only	Serum, stool	1. Number of bacterial spp. (*p* = 0.01) and diversity (*p* = 0.004) significantly increased only with intervention. 2. Bacteroidetes:Firmicutes ratio decreased with intervention and increased in controls (*p* = 0.004). 3. Significant improvement in metabolic and anthropometric parameters (BW, BMI, glucose, and insulin) compared with Mediterranean diet alone
10	Lahiji, 2021a [50]	Overweight or obese postmenopausal BC survivors	LCD + 10^9^ CFU/day of synbiotics for 8 weeks	LCD + Placebo	Serum	1. Insignificant reducing effects on glycemic profile (serum insulin, fasting plasma glucose, HbA1c, HOMA-IR), IGF-1, and sex hormones (estradiol, testosterone, DHEA-S, and SHBG).
11	Lahiji, 2021b [51]					1. Increased adiponectin (*p* < 0.001), reduced TNF-α (*p* < 0.001) and hs-CRP (*p* < 0.001) compared to placebo.
12	Juan, 2022 [52]	BC patients who underwent 4 cycles of docetaxel-based chemotherapy	Twice daily, 3 capsules (0.84 g)/time of probiotics during chemotherapy at a cycle of 21 d for a total of four cycles	Placebo	Plasma, stool	1. Supplement significantly decreased the CRCI, improved the allover cognitive functions, changed gut microbial, and modulated 9 plasma metabolite changes. 2. Metabolites *p*-mentha-1,8-dien-7-ol, linoelaidyl carnitine, and 1-aminocyclopropane-1-carboxylic acid negatively correlated with rate of CRCI.
13	Juan, 2021 [53]					1. *Bacteroides* (*p* < 0.001) and *Anaerostipes* (*p* < 0.001) changes inversely correlated with change in LDL. 2. Reduced BW, BF%, and LDL, and minimized metabolic changes and gut dysbacteriosis.

**Table 4:** Quantitative subgroup analysis for all the included trials.

*Subgroup/Sensitivity Analysis*		*Number of Trials*	*SMD (95% CI)*	*p-Value*	*Heterogeneity (I^2^, p-Value)*
***BMI***					
*Probiotics ± prebiotics*	Probiotics only	2	0.00 (−0.76, 0.77)	0.99	73% (0.05)
	Combined with FOS	3	−0.05 (−0.29, 0.20)	0.72	0% (0.99)
*Intake duration*	10 weeks	3	−0.06 (−0.30, 0.19)	0.65	0% (1.00)
	8 weeks	2	0.14 (−0.30, 0.58)	0.53	19% (0.27)
	3 weeks	1	−0.34 (−0.75, 0.07)	0.11	N/A
***Body weight***					
*Probiotics ± prebiotics*	Probiotics only	2	0.10 (−1.08, 1.28)	0.87	88% (0.004)
	Combined with FOS	2	−0.01 (−0.32, 0.30)	0.93	0% (0.54)
*Intake duration*	10 weeks	1	0.08 (−0.34, 0.49)	0.73	N/A
	8 weeks	2	0.27 (−0.57, 1.10)	0.53	75% (0.04)
	3 weeks	1	−0.47 (−0.88, −0.06)	0.03	N/A
***BF%***					
*Probiotics ± prebiotics*	Probiotics only	1	−0.51 (−0.93, −0.09)	0.02	N/A
	Combined with FOS	2	−0.03 (−0.34, 0.28)	0.85	0% (0.86)
*Intake duration*	10 weeks	1	−0.00 (−0.42, 0.41)	0.98	N/A
	8 weeks	1	−0.06 (−0.52, 0.40)	0.80	N/A
	3 weeks	1	−4.50 (−5.28, −3.72)	<0.00001	N/A
***Waist circumference***					
*Probiotics ± prebiotics*	Probiotics only	1	4.0 (−1.44, 9.44)	0.15	N/A
	Combined with FOS	2	−1.10 (−4.52, 2.31)	0.53	0% (0.84)
*Intake duration*	10 weeks	1	−0.14 (−0.56, 0.28)	0.36	0% (1.00)
	8 weeks	2	0.19 (−0.24, 0.63)	0.39	18% (0.27)

Abbreviations: BMI, body mass index; BF%, percentage change in body fat; FOS, fructo-oligosaccharides; N/A, not available.

## Data Availability

Data will be available as Supplementary Files.

## Supplementary Materials

The following supporting information can be downloaded at: https://www.mdpi.com/article/10.3390/biology12020280/s1, Supplementary Information File S1—Table S1: PRISMA 2020 Checklist [65]; Supplementary Information File S2—Table S2: Full search strategy.

## Abbreviations

CENTRAL: Cochrane Central Register of Controlled Trials; CFU: colony-forming units; CI: confidence intervals; GRADE: Grading of Recommendation Assessment, Development and Evaluation; MD: mean differences; PRISMA-P: Preferred Reporting Items for Systematic Review and Meta-Analysis Protocols; RCTs: randomized controlled trials.
