# Correction: Thu et al. Effect of Probiotics in Breast Cancer: A Systematic Review and Meta-Analysis. *Biology* 2023, *12*, 280

**DOI:** 10.3390/biology14080991

**Published:** 2025-08-04

**Authors:** May S. Thu, Thunnicha Ondee, Tanawin Nopsopon, Izzati A. K. Farzana, Joanne L. Fothergill, Nattiya Hirankarn, Barry J. Campbell, Krit Pongpirul

**Affiliations:** 1 Center of Excellence in Immunology and Immune-Mediated Diseases, Department of Microbiology, Faculty of Medicine, Chulalongkorn University, Bangkok 10330, Thailand; mst.maysoethu@gmail.com (M.S.T.);; 2 Department of Infection Biology & Microbiomes, Institute of Infection, Veterinary and Ecological Sciences, University of Liverpool, Liverpool L69 3GE, UK; 3 Joint Chulalongkorn University—University of Liverpool PhD Programme in Biomedical Sciences and Biotechnology, Faculty of Medicine, Chulalongkorn University, Bangkok 10330, Thailand; 4 Department of Preventive and Social Medicine, Faculty of Medicine, Chulalongkorn University, Bangkok 10330, Thailand; 5 School of Global Health, Faculty of Medicine, Chulalongkorn University, Bangkok 10330, Thailand; 6 Harvard T.H. Chan School of Public Health, Harvard University, Boston, MA 02215, USA; 7 Department of Clinical Infection, Microbiology & Immunology, Institute of Infection, Veterinary and Ecological Sciences, University of Liverpool, Liverpool L69 7BE, UK; 8 Department of International Health, Johns Hopkins Bloomberg School of Public Health, Baltimore, MD 21211, USA; 9 Bumrungrad International Hospital, Bangkok 10110, Thailand

## Text Correction

There was an error in the original publication [1]. Data was incorrectly extracted from Table 3 of Juan et al. [53], where we had taken the value for % body fat in the post-probiotic group and compared it to body weight in the post-placebo group. Data has been reanalyzed using the correct value for % body fat in the post-placebo group.

A correction has been made to Section 3.8.

### 3.8. Percentage Change in Body Fat

Three studies assessed percentage change in body fat (BF%) after probiotics intervention. Use of probiotics reduced BF% in both breast cancer patients and survivors (MD = −1.81; 95% CI: −5.22 to 1.61; *p* = 0.30; Figure 4A). Subgroup analysis demonstrated that ProLBE supplements significantly reduced the elevation of BF% in breast cancer patients (MD = −4.20; 95% CI: −7.59 to −0.81; *p* = 0.02) while a smaller decrease in BF% occurred in breast cancer survivors with use of ProLBS capsules (Figure 4B).

As such, a correction has also been made to Table 4 and Figure 4 to reflect the revised analysis.

The corrected Table 4 appears below:

**Table 4:** Quantitative subgroup analysis for all the included trials.

*Subgroup/Sensitivity Analysis*		*Number of Trials*	*SMD (95% CI)*	*p-Value*	*Heterogeneity (I^2^, p-Value)*
***BMI***					
*Probiotics ± prebiotics*	Probiotics only	2	0.00 (−0.76, 0.77)	0.99	73% (0.05)
	Combined with FOS	3	−0.05 (−0.29, 0.20)	0.72	0% (0.99)
*Intake duration*	10 weeks	3	−0.06 (−0.30, 0.19)	0.65	0% (1.00)
	8 weeks	2	0.14 (−0.30, 0.58)	0.53	19% (0.27)
	3 weeks	1	−0.34 (−0.75, 0.07)	0.11	N/A
***Body weight***					
*Probiotics ± prebiotics*	Probiotics only	2	0.10 (−1.08, 1.28)	0.87	88% (0.004)
	Combined with FOS	2	−0.01 (−0.32, 0.30)	0.93	0% (0.54)
*Intake duration*	10 weeks	1	0.08 (−0.34, 0.49)	0.73	N/A
	8 weeks	2	0.27 (−0.57, 1.10)	0.53	75% (0.04)
	3 weeks	1	−0.47 (−0.88, −0.06)	0.03	N/A
***BF%***					
*Probiotics ± prebiotics*	Probiotics only	1	−0.51 (−0.93, −0.09)	0.02	N/A
	Combined with FOS	2	−0.03 (−0.34, 0.28)	0.85	0% (0.86)
*Intake duration*	10 weeks	1	−0.00 (−0.42, 0.41)	0.98	N/A
	8 weeks	1	−0.06 (−0.52, 0.40)	0.80	N/A
	3 weeks	1	−4.50 (−5.28, −3.72)	<0.00001	N/A
***Waist circumference***					
*Probiotics ± prebiotics*	Probiotics only	1	4.0 (−1.44, 9.44)	0.15	N/A
	Combined with FOS	2	−1.10 (−4.52, 2.31)	0.53	0% (0.84)
*Intake duration*	10 weeks	1	−0.14 (−0.56, 0.28)	0.36	0% (1.00)
	8 weeks	2	0.19 (−0.24, 0.63)	0.39	18% (0.27)

Abbreviations: BMI, body mass index; BF%, percentage change in body fat; FOS, fructo-oligosaccharides; N/A, not available.

The corrected Figure 4 appears below:

**Figure 4:**
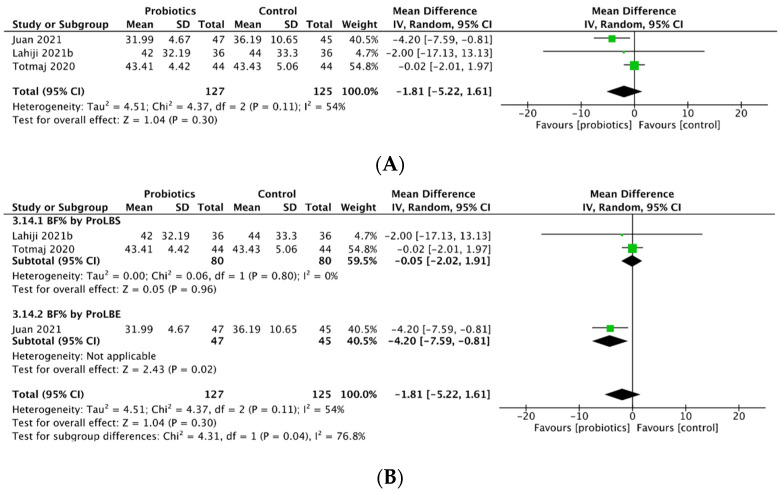
Meta-analysis for (**A**) percentage change in body fat (BF%) percent and (**B**) BF% by probiotic type.

The authors state that the scientific conclusions are unaffected. This correction was approved by the Academic Editor. The original publication has also been updated.
