# Supplementary material for: Effect of Probiotics in Breast Cancer: A Systematic Review and Meta-Analysis
Source: Biology (Basel). 2023 Feb 9;12(2):280. doi: 10.3390/biology12020280 (PMC10004677; doi:10.3390/biology12020280)
Supplement: Supplementary file 1 [file biology-12-00280-s001.zip › S2_Full search strategy_PBBC_v1.4.pdf]

Effect of Probiotics in Breast Cancer: A Systematic Review and Meta-Analysis

May S. Thu, Thunnicha Ondee, Tanawin Nopsopon, Izzati A.K. Farzana, Joanne L. Fothergill, Nattiya Hirankarn, Barry J. Campbell and Krit Pongpirul

Supplementary Information file S2. Full search strategy

Set #	Pubmed	Results
1 Probiotics	"probiotics"[MeSH Terms] OR probiotic*[tiab] OR “prebiotics”[MeSH Terms] OR prebiotics*[tiab] OR "synbiotics"[MeSH Terms] OR synbiotic*[tiab] OR “gastrointestinal microbiome”[MeSH Terms] OR "lactobacillales"[MeSH Terms] OR “lactobacillus”[MeSH Terms] OR “Saccharomyces boulardii”[MeSH Terms] OR lactobacill*[tiab] OR pediococcus[tiab] OR enterococcus[tiab] OR leuconostoc[tiab] OR oenococcus[tiab] OR weissella[tiab] OR lactococcus[tiab] OR streptococcus[tiab] OR Escherichia[tiab] OR "bifidobacterium"[MeSH Terms] OR bifidobacteri*[tiab] OR aeriscardovia[tiab] OR alloscardovia[tiab] OR bombiscardovia[tiab] OR galliscardovia[tiab] OR neoscardovia[tiab] OR parascardovia[tiab] OR pseudoscardovia[tiab] OR scardovia[tiab] OR "gardnerella"[MeSH Terms] OR gardnerella[tiab] OR "propionibacterium"[MeSH Terms] OR propionibacteri*[tiab] OR "staphylococcus"[MeSH Terms] OR staphylococcus[tiab] OR "bacillales"[MeSH Terms] OR bacillus[tiab] OR bacill*[tiab] OR "saccharomyces"[MeSH Terms] OR saccharomyces[tiab] OR ((“alimentary canal”[tiab] OR “alimentary tract”[tiab] OR “digestive tract”[tiab] OR enteric[tiab] OR gastrointestin*[tiab] OR gut[tiab] OR gastric[tiab] OR intestin*[tiab]) AND (flor*[tiab] OR microb*[tiab] OR microflor*[tiab] OR bacteri*[tiab] OR microorganism[tiab])) OR “L. acidophilus”[tiab] OR “L. brevis”[tiab] OR “L. casei”[tiab] OR “L. plantarum”[tiab] OR “L. reuteri”[tiab] OR “L. delbrueckii”[tiab] OR “L. fermentum”[tiab] OR “L. gasseri”[tiab] OR “L. helveticus”[tiab] OR “L. johnsonii”[tiab] OR “L. reuteri”[tiab] OR “L. rhamnosus”[tiab] OR “L. salivarius”[tiab] OR “L. sporogenes”[tiab] OR “B. animalis”[tiab] OR “B. bifidum”[tiab] OR “B. breve”[tiab] OR “B. longum”[tiab] OR “B. infantis”[tiab] OR “S. thermophilus”[tiab] OR “E. faecium”[tiab] OR “E. durans”[tiab] OR “P. acidilactici”[tiab] OR “L. mesenteroides”[tiab] OR “B. coagulans”[tiab] OR “B. subtilis”[tiab] OR “B. cereus”[tiab] OR "VSL #3"[tiab] OR “lactobacillus GG”[tiab] OR “acidophilus”[tiab] OR “Bacid”[tiab] OR “Lactinex”[tiab] OR “Culturelle”[tiab] OR “Florastor”[tiab] OR “Flora-Q”[tiab] OR “Florajen3”[tiab] OR “bulgaricus”[tiab] OR “paracasei”[tiab] OR “lactis”[tiab] OR “plantarum”[tiab] OR “bifidum”[tiab] OR “breve”[tiab] OR “lactis”[tiab] OR “longum”[tiab] OR “thermophilus”[tiab]	**966,565**
2 Breast Cancer	"breast neoplasms"[MeSH Terms] OR (breast[tiab] AND (cancer*[tiab] OR neoplas*[tiab] OR tumor*[tiab] OR tumour*[tiab] OR malignan*[tiab] OR carcinoma*[tiab] OR adenocarcinoma*[tiab] OR "metastasis"[tiab]))	**450,425**
3 RCT	“randomized controlled trial”[pt] OR “controlled clinical trial”[pt] OR “pragmatic clinical trial”[pt] OR random*[tiab] OR placebo[tiab] OR "drug therapy"[sh] OR trial[tiab] OR groups[tiab] OR RCTs*[tiab]	**5,556,165**
4	#1 AND #2 AND #3	**553**
5	animals[MeSH Terms] NOT humans[MeSH Terms]	**4,942,886**
6	#4 NOT #5	**516**
7	English[lang]	**28,785,738**
8	#6 AND #7	**487**

Set #	CENTRAL	Results
1<br>Probiotics	[mh "probiotics"] OR probiotic*:ti,ab,kw OR [mh “prebiotics”] OR prebiotics*:ti,ab,kw OR [mh "synbiotics"] OR synbiotic*:ti,ab,kw OR lactobacill*:ti,ab,kw OR pediococcus:ti,ab,kw OR enterococcus:ti,ab,kw OR leuconostoc:ti,ab,kw OR oenococcus:ti,ab,kw OR weissella:ti,ab,kw OR lactococcus:ti,ab,kw OR streptococcus:ti,ab,kw OR [mh "bifidobacterium"] OR bifidobacteri*:ti,ab,kw OR aeriscardovia:ti,ab,kw OR alloscardovia:ti,ab,kw OR bombiscardovia:ti,ab,kw OR galliscardovia:ti,ab,kw OR neoscardovia:ti,ab,kw OR parascardovia:ti,ab,kw OR pseudoscardovia:ti,ab,kw OR scardovia:ti,ab,kw OR [mh "gardnerella"] OR gardnerella:ti,ab,kw OR [mh "propionibacterium"] OR propionibacteri*:ti,ab,kw OR [mh "staphylococcus"] OR staphylococcus:ti,ab,kw OR [mh "bacillales"] OR bacillus:ti,ab,kw OR bacill*:ti,ab,kw OR [mh "saccharomyces"] OR saccharomyces:ti,ab,kw OR ((“alimentary canal”:ti,ab,kw OR “alimentary tract”:ti,ab,kw OR “digestive tract”:ti,ab,kw OR enteric:ti,ab,kw OR gastrointestin*:ti,ab,kw OR gut:ti,ab,kw OR gastric:ti,ab,kw OR intestin*:ti,ab,kw) AND (flor*:ti,ab,kw OR microb*:ti,ab,kw OR microflor*:ti,ab,kw OR bacteri*:ti,ab,kw OR microorganism:ti,ab,kw)) OR “L. acidophilus”:ti,ab,kw OR “L. brevis”:ti,ab,kw OR “L. casei”:ti,ab,kw OR “L. delbrueckii”:ti,ab,kw OR “L. fermentum”:ti,ab,kw OR “L. gasseri”:ti,ab,kw OR “L. helveticus”:ti,ab,kw OR “L. johnsonii”:ti,ab,kw OR “L. reuteri”:ti,ab,kw OR “L. rhamnosus”:ti,ab,kw OR “L. salivarius”:ti,ab,kw OR “L. sporogenes”:ti,ab,kw OR “L. plantarum”:ti,ab,kw OR “L. reuteri”:ti,ab,kw OR “B. infantis”:ti,ab,kw OR “B. animalis”:ti,ab,kw OR “B. bifidum”:ti,ab,kw OR “B. breve”:ti,ab,kw OR “B. longum”:ti,ab,kw OR “S. boulardi”:ti,ab,kw OR “L. lactis”:ti,ab,kw OR “E. durans”:ti,ab,kw OR “E. faecium”:ti,ab,kw OR “S. thermophilus”:ti,ab,kw OR “P. acidilactici”:ti,ab,kw OR “L. mesenteroides”:ti,ab,kw OR “B. coagulans”:ti,ab,kw OR “B. subtilis”:ti,ab,kw OR “B. cereus”:ti,ab,kw OR “gastrointestinal microbiome”:ti,ab,kw OR lactobacillales:ti,ab,kw OR lactobacillus:ti,ab,kw OR acidophilus:ti,ab,kw OR Bacid:ti,ab,kw OR Lactinex:ti,ab,kw OR Culturelle:ti,ab,kw OR Florastor:ti,ab,kw OR “Flora-Q”:ti,ab,kw OR Floranex:ti,ab,kw OR “Flora-Q2”:ti,ab,kw OR Florajen3:ti,ab,kw OR “Probiotic 123”:ti,ab,kw	**30427**
2<br>Breast<br>Cancer	[mh "breast neoplasms"] OR (breast:ti,ab,kw AND (cancer*:ti,ab,kw OR neoplas*:ti,ab,kw OR tumor*:ti,ab,kw OR tumour*:ti,ab,kw OR malignan*:ti,ab,kw OR carcinoma*:ti,ab,kw OR adenocarcinoma*:ti,ab,kw OR metastasis*:ti,ab,kw))	**41474**
3	#1 AND #2	**186**

Set #	**Embase**	Results
1 Probiotics	'probiotic agent'/exp OR 'probiotic*':ti,ab OR 'prebiotic agent'/exp OR 'prebiotic*':ti,ab OR 'synbiotic agent'/exp OR 'synbiotic*':ti,ab OR 'intestine flora'/exp OR 'lactobacillales'/exp OR 'lactobacillus'/exp OR 'lactobacill*':ti,ab OR 'pediococcus':ti,ab OR 'enterococcus':ti,ab OR 'leuconostoc':ti,ab OR 'oenococcus':ti,ab OR 'weissella':ti,ab OR 'lactococcus':ti,ab OR 'streptococcus':ti,ab OR 'bifidobacterium'/exp OR 'bifidobacteri*':ti,ab OR 'aeriscardovia':ti,ab OR 'alloscardovia':ti,ab OR 'bombiscardovia':ti,ab OR 'galliscardovia':ti,ab OR 'neoscardovia':ti,ab OR 'parascardovia':ti,ab OR 'pseudoscardovia':ti,ab OR 'scardovia':ti,ab OR 'gardnerella'/exp OR 'gardnerella':ti,ab OR 'propionibacterium'/exp OR 'propionibacteri*':ti,ab OR 'staphylococcus'/exp OR 'staphylococcus':ti,ab OR 'bacillales'/exp OR 'bacillus':ti,ab OR 'bacill*':ti,ab OR 'saccharomyces'/exp OR 'saccharomyces':ti,ab OR (('alimentary canal':ti,ab OR 'alimentary tract':ti,ab OR 'digestive tract':ti,ab OR 'enteric':ti,ab OR 'gastrointestin*':ti,ab OR 'gut':ti,ab OR 'gastric':ti,ab OR 'intestin*':ti,ab) adj6 ('flor*':ti,ab OR 'microb*':ti,ab OR 'microflor*':ti,ab OR 'bacteri*':ti,ab OR 'microorganism':ti,ab)) OR 'L. acidophilus':ti,ab OR 'L. brevis':ti,ab OR 'L. casei':ti,ab OR 'L.delbrueckii':ti,ab OR 'L. fermentum':ti,ab OR 'L. gasseri':ti,ab OR 'L. helveticus':ti,ab OR 'L. johnsonii':ti,ab OR 'L. reuteri':ti,ab OR 'L. rhamnosus':ti,ab OR 'L. salivarius':ti,ab OR 'L. sporogenes':ti,ab OR 'L. plantarum':ti,ab OR 'L. infantis':ti,ab OR 'S. boulardi':ti,ab OR 'B. animalis':ti,ab OR 'B. bifidum':ti,ab OR 'B. breve':ti,ab OR 'B. longum':ti,ab OR 'E. faecium':ti,ab OR 'S. thermophilus':ti,ab OR 'P. acidilactici':ti,ab OR 'L. mesenteroides':ti,ab OR 'B. coagulans':ti,ab OR 'B. subtilis':ti,ab OR 'B. cereus':ti,ab OR 'Bacid':ti,ab OR 'Lactinex':ti,ab OR 'Culturelle':ti,ab OR 'Florastor':ti,ab OR 'Flora-Q':ti,ab OR 'Flora-Q2':ti,ab OR 'Floranex':ti,ab OR 'Florajen3':ti,ab OR 'Probiotic 123':ti,ab	
2 Breast Cancer	'breast cancer'/exp OR ('breast':ti,ab AND ('cancer*':ti,ab OR 'neoplas*':ti,ab OR 'tumor*':ti,ab OR 'tumour*':ti,ab OR 'malignan*':ti,ab OR 'carcinoma*':ti,ab OR 'adenocarcinoma*':ti,ab))	
3 RCTs	'randomized controlled trial'/exp OR 'controlled clinical trial'/exp OR 'controlled study'/exp OR 'double blind procedure'/exp OR 'single blind procedure'/exp OR 'crossover procedure'/exp OR 'placebo'/exp OR 'randomization'/exp OR 'random*':ti,ab OR 'placebo?':ti,ab OR 'allocat*':ti,ab OR 'assign*':ti,ab OR 'blind*':ti,ab OR 'cross-over':ti,ab OR 'crossover':ti,ab OR 'compare':ti,ab OR 'compared':ti,ab OR 'comparing':ti,ab OR 'comparison':ti,ab OR 'comparative':ti,ab OR ('controlled':ti,ab adj7 'study':ti,ab) OR ('controlled':ti,ab adj7 'design':ti,ab) OR ('singl*':ti,ab adj7 'mask*':ti,ab) OR ('doubl*':ti,ab adj7 'mask*':ti,ab) OR ('trebl*':ti,ab adj7 'mask*':ti,ab) OR ('tripl*':ti,ab adj7 'mask*':ti,ab) OR 'trial':ti,ab	
4	#1 AND #2 AND #3	
5	[animals]/lim NOT [humans]/lim	
6	#4 NOT #5	
